# Modelling the Effect of Matrix Metalloproteinases in Dermal Wound Healing

**DOI:** 10.1007/s11538-023-01195-8

**Published:** 2023-09-05

**Authors:** Sonia Dari, Nabil T. Fadai, Reuben D. O’Dea

**Affiliations:** School of Mathematical Sciences, University of Nottingham, Nottingham, NG7 2RD UK

**Keywords:** Travelling waves and Bifurcation analysis

## Abstract

With over 2 million people in the UK suffering from chronic wounds, understanding the biochemistry and pharmacology that underpins these wounds and wound healing is of high importance. Chronic wounds are characterised by high levels of matrix metalloproteinases (MMPs), which are necessary for the modification of healthy tissue in the healing process. Overexposure of MMPs, however, adversely affects healing of the wound by causing further destruction of the surrounding extracellular matrix. In this work, we propose a mathematical model that focuses on the interaction of MMPs with dermal cells using a system of partial differential equations. Using biologically realistic parameter values, this model gives rise to travelling waves corresponding to a front of healthy cells invading a wound. From the arising travelling wave analysis, we observe that deregulated apoptosis results in the emergence of chronic wounds, characterised by elevated MMP concentrations. We also observe hysteresis effects when both the apoptotic rate and MMP production rate are varied, providing further insight into the management (and potential reversal) of chronic wounds.

## Introduction

Wound healing is a physiological response to injury of tissue involving the coordinated interactions of many cell types and biochemical agents (Wallace et al. 2021; Martin 1997). In individuals such as diabetes patients, so-called ‘chronic wounds’ may persist, which require medical treatments to promote healing (Nyugen et al. 2016; Fan et al. 2021). There are approximately 2.2 million people in the UK suffering from chronic wounds, costing the NHS over $\pounds 5$ billion per year (Department of Health and Social Care 2021). These wounds last, on average, 12–13 months and recur in $60\%$–$70\%$ of individuals, potentially leading to the loss of function (Frykberg and Banks 2015). Improved understanding of the biochemical mechanisms underpinning chronic wounds is therefore crucial to support the development of new treatments.

The healing of wounds involves the complex interplay of various cell types and their mediators and cytokines (Flegg et al. 2015). A wound is defined as damage to the skin which is comprised of two layers: the epidermis and the dermis. The epidermis is the outermost layer and is responsible for protecting against infection, while the dermis is the innermost layer and provides tensile strength for the skin by means of the dermal extracellular matrix (ECM) (Flegg et al. 2015; Frantz et al. 2010). The process of wound healing can be categorised into four successive stages: haemostasis, inflammation, proliferation and remodelling (Wallace et al. 2021). A wound that is able to complete these four stages in a well-coordinated manner is defined as an acute wound; conversely, a wound that spends a prolonged time in any of the four stages is defined as a chronic wound (Nyugen et al. 2016). The inflammation and proliferation stages of wound healing involve the production of matrix metalloproteinases (MMPs) by many cells types such as keratinocytes, fibroblasts, endothelial cells, and inflammatory cells (Guo and DiPietro 2010; Nyugen et al. 2016). For the remainder of this work, we consider only the production of MMPs by fibroblasts. MMPs are responsible for lysing protein components of the ECM, allowing for fibroblast migration within the ECM and, as a result, the proliferation of cells and modification of tissue (Nyugen et al. 2016). Experimental data has shown that there is increased expression of MMPs at the edge of a wound in all stages of the healing of an acute wound (Krejner et al. 2016). It is essential to have a well regulated concentration of MMPs during this process: if MMP levels are too low, this leads to uncontrolled ECM production and can cause issues such as hypertrophic scarring or dermal fibrosis (Li et al. 2009). If MMP levels are too high, chronic wounds are observed, as the overexposure of MMPs results in the increased degradation of the ECM (Sabino and Keller 2015). One such cause for these elevated MMP levels is deregulated apoptosis of dermal tissue, which may be caused by uncontrolled blood sugar levels in diabetes patients (Rai et al. 2005; Arya et al. 2014).

Mathematical modelling of the wound healing process can provide a framework to understand the behaviour of chronic wounds, potentially to direct treatments and improve the quality of life of patients. Many mathematical models have been developed to describe the wound healing process, each focusing on specific mechanisms of interest. For example, agent–based models (ABMs) can emulate the individual-level stochastic nature of the biological processes involved in wound healing (Sun et al. 2009; Mi et al. 2007; Walker et al. 2004; Ziraldo et al. 2013; An et al. 2009; Bankes 2002). Although ABMs are able to detail specific properties of individual cells, using them to model dynamics of wound healing on a tissue level may be computationally infeasible. We therefore will consider continuous models of wound healing, which typically use partial differential equations (PDEs) and present opportunities for a range of analytical techniques for further study (Flegg et al. 2015).

Continuous mathematical models of both dermal and epidermal wound healing typically take the form of reaction-diffusion systems, extending the work of Sherratt and Murray (1990) that examined the interaction of epidermal cells with a representative chemical acting as a regulator of mitosis. Their model has subsequently been developed to consider additional chemical agents involved in epidermal wound healing, such as the role of epidermal growth factor in corneal wound healing and the role of keratinocyte growth factor in epidermal wound healing (Sherratt and Murray 1992; Dale et al. 1994; Sheardown and Cheng 1996; Gaffney et al. 1999). Contrastingly, other models of dermal wound healing consider the role of fibroblasts in restoring the ECM in response to various mechanisms at different stages in the wound healing process. Many of these models consider the roles of angiogenesis and consider the role of oxygen transport in wound healing (Landen et al. 2016; Chaplain and Byrne 1996; Pettet et al. 1996a, b; Schugart et al. 2008; Flegg et al. 2015), while others consider the process of ECM restoration by fibroblasts in response to growth factors (Dale et al. 1997; Wearing and Sherratt 2000), the crosstalk of the epidermis and the dermis to simulate the simultaneous healing of both layers (Menon et al. 2012; Menon and Flegg 2021) and the incorporation of collagen fibre orientation due to cell movement (Dallon et al. 1999, 2000, 2001; McDougall et al. 2006; Cumming et al. 2009). Travelling wave solutions are often observed in these mathematical models, agreeing with the experimental evidence of wound healing assays (Maini et al. 2004a, b). However, these models are often limited to the healing of acute wounds.

In this work, we consider the role that MMPs play in wound healing, with particular consideration given to chronic wounds. We propose a two-component reaction-diffusion model that describes the interaction of MMPs with dermal tissue, in which the restoration of the ECM is affected by MMP concentration levels. Using biologically realistic parameter values, this model gives rise to travelling waves corresponding to a front of healthy cells invading a wound. From the arising travelling wave analysis, we observe that deregulated apoptosis results in the emergence of chronic wounds, characterised by elevated MMP concentrations. We also observe hysteresis effects when both the apoptotic rate and MMP production rate are varied, providing further insight into the management (and potential reversal) of chronic wounds.

We develop our mathematical model in Sect. 2. Direct numerical simulations indicate the existence of travelling wave solutions, motivating a travelling wave analysis in Sect. 3. We then consider the effect of deregulated apoptosis on the healing of a wound via bifurcation and phase plane analysis in Sect. 4. Finally, we consider the effects of elevated MMP production levels on the healing of a wound in Sect. 6, before discussing our results in Sect. 7.

## Model Development

We begin by presenting a mathematical formulation to describe MMP concentration dynamics in the presence of a wound. Elevated levels of MMPs play a key role in the persistence of chronic wounds; in light of this, we focus on constructing a reaction-diffusion model of wound healing that incorporates the interaction between MMPs with dermal cells during the wound healing process. For simplicity, we consider a wound with a one-dimensional Cartesian geometry, where *x* represents the longitudinal distance across a fixed domain, which evolves over time *t*. This model consists of two populations within a wound: the dermal cell density, denoted as *n*(*x*, *t*), and the MMP concentration, denoted as *m*(*x*, *t*). We take *n*(*x*, *t*) to incorporate the actions of both fibroblasts and ECM for the sake of simplicity, since fibroblasts are responsible for creating the components of the ECM.

We assume that dermal cells undergo mitosis with intrinsic growth rate $\sigma$ and carrying capacity $K_n$, noting that $n=0$ corresponds to the complete absence of cell tissue. As mentioned previously, MMPs can assist in the healing of the wound up to a threshold concentration, denoted here as $m_\text {thresh}$, above which they adversely affect healing of the wound by degrading the surrounding ECM. This effect is incorporated in the mitosis process by means of a function *f*(*m*). Furthermore, dermal cells undergo apoptosis with rate $\delta _n$ and dermal cell motion within the wound is represented by linear diffusion with effective diffusivity $D_n$.

**Fig. 1:**
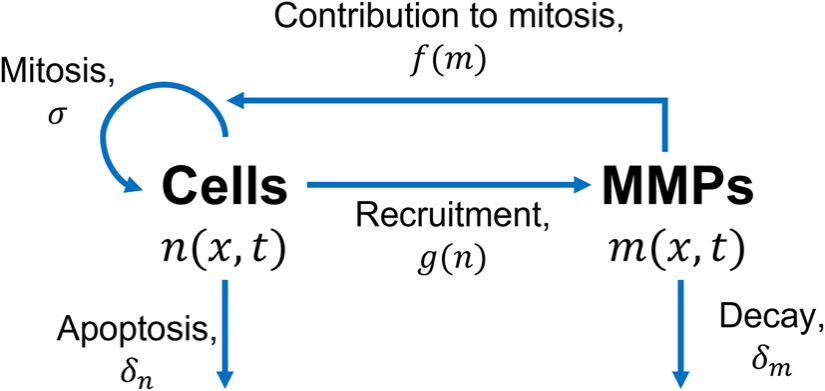
Schematic of the interactions of dermal cells with MMPs. Labelled rates are defined as in the system (1), (2)

MMP production by fibroblasts, denoted by a recruitment function *g*(*n*), occurs in response to inflammatory chemicals which are found in abundance in a wound. MMP production is therefore assumed here to be proportional to the absence of healthy tissue. MMPs undergo natural decay with rate $\delta _m$ and diffuse through the wound with diffusivity $D_m$. These aforementioned processes are shown in Fig. 1. Combining these modelling elements provides the following system of PDEs:

1 $$\begin{aligned}{} & {} \frac{\partial n}{\partial t} = \underbrace{D_n \frac{\partial ^2 n}{\partial x^2}}_{\begin{array}{c} \text {Cell} \\ \text {motion} \end{array}} + \underbrace{\sigma \big (1 + f(m)\big )n\bigg (1-\frac{n}{K_n}\bigg )}_\text {Mitosis} - \underbrace{\delta _n n}_\text {Apoptosis}, \end{aligned}$$

2 $$\begin{aligned}{} & {} \frac{\partial m}{\partial t} = \underbrace{D_m \frac{\partial ^2 m}{\partial x^2}}_\text {Diffusion} + \underbrace{g(n)}_{\begin{array}{c} \text {Recruitment} \\ \text { of { m}} \end{array}} - \underbrace{\delta _m m}_\text {Decay}. \end{aligned}$$

**Fig. 2:**
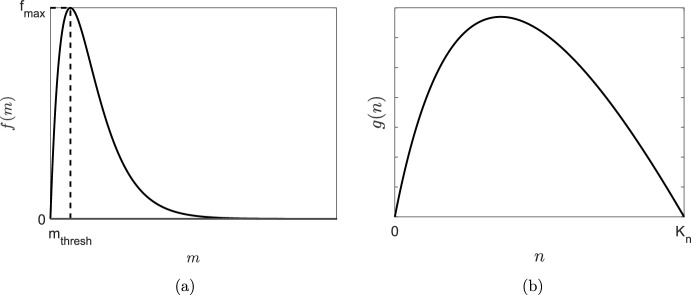
Schematic functional forms of **a** the healing contribution by MMPs function, *f*(*m*) in (3), and **b** the MMP recruitment function, *g*(*n*) in (4)

We now discuss the functional forms of *f*(*m*) and *g*(*n*) appearing in the system (1), (2). The function *f*(*m*) in (1) represents the contribution to wound healing by MMPs and is defined to be an increasing function of *m* until $m=m_\text {thresh}$, at which $f(m)=f_\text {max}$, the maximum factor by which mitosis can be enhanced due to the presence of MMPs. Larger concentrations of MMPs hinder the wound healing process and thus, for *m* larger than $m_\text {thresh}$, *f*(*m*) is a decreasing function of *m*. A suitable choice of *f*(*m*) is therefore

3 $$\begin{aligned} f(m) = \frac{f_\text {max}}{m_\text {thresh}} m \text { exp}\bigg (1 - \frac{m}{m_\text {thresh}}\bigg ); \end{aligned}$$

a schematic of *f*(*m*) is shown in Fig. 2a. We note that *f*(*m*) and hence the parameter $f_\text {max}$ are dimensionless. For elevated levels of MMPs, *f*(*m*) decreases to zero and the mitosis term reduces to logistic growth with zero healing contribution due to MMPs. One could also allow *f*(*m*) to take negative values for large *m*, representing a negative healing contribution to the ECM. We have considered a functional form of this type in Appendix A, where we show that such a choice gives rise to largely similar qualitative features to those produced by the functional form of *f*(*m*) given in (3). Given these similarities, we restrict attention to the choice of *f*(*m*) as in (3) as this choice results in simpler analytic computations.

The recruitment function *g*(*n*) in (2) represents the production of MMPs by the cells in proportion to the absence of healthy tissue. Consequently, we can represent *g*(*n*) as

4 $$\begin{aligned} g(n) = \bigg (1-\frac{n}{K_n}\bigg )\frac{k_m n}{\gamma _m + n}, \end{aligned}$$

where $k_m$ is the maximum reaction rate and $\gamma _m$ is a Michaelis–Menten constant (Michaelis and Menten 1913). A schematic of *g*(*n*) is shown in Fig. 2b.

### Initial Conditions and Boundary Conditions

The initial conditions of the system (1), (2) are chosen as follows to emulate a wound starting at $x=\frac{\lambda }{\nu }$, i.e the tissue surrounding the wound occupies the spatial interval $[0,\frac{\lambda }{\nu })$, and the wound is the region $[\frac{\lambda }{\nu }, L]$. Furthermore, a small concentration of MMPs, $\eta$, is initially present at the wound edge $\frac{\lambda }{\nu }$:

5 $$\begin{aligned}{} & {} n(x,0) = K_n H(\lambda - \nu x), \end{aligned}$$

6 $$\begin{aligned}{} & {} m(x,0)= \eta \text { exp}\big (-(\nu x-\lambda )^2\big ). \end{aligned}$$

In (5), *H* is the Heaviside function while $n=K_n$ corresponds to the cells being at carrying capacity. Assuming the wound to be symmetric about its centre and that the tissue is fully healed far away from the wound, we adopt zero-flux boundary conditions on both endpoints of the finite domain of length *L*, i.e:

7 $$\begin{aligned} \frac{\partial }{\partial x}n(x,t) = \frac{\partial }{\partial x}m(x,t) = 0 \quad \text {at} \quad x = 0,L. \end{aligned}$$

### Nondimensionalisation

In this section, we non-dimensionalise the system (1)–(7). We are interested in the dynamics of the system on the timescale of mitotic rate of dermal cells and therefore introduce the following dimensionless variables:

8 $$\begin{aligned} N = \frac{n}{K_n}, \qquad M = \frac{m}{m_\text {thresh}}, \qquad X = \sqrt{\frac{\sigma }{D_n}} x, \qquad T = \sigma t. \end{aligned}$$

With these scalings, we obtain the following dimensionless equations:

9 $$\begin{aligned}{} & {} \frac{\partial N}{\partial T} = \frac{\partial ^2 N}{\partial X^2} + \big [1 + f_\text {max} M \text {exp}(1-M)\big ] N (1-N) - \delta _N N, \end{aligned}$$

10 $$\begin{aligned}{} & {} \frac{\partial M}{\partial T} = D_M\frac{\partial ^2 M}{\partial X^2} + \frac{\alpha N(1-N)}{\gamma _M+N} - \delta _M M, \end{aligned}$$

where

11 $$\begin{aligned} D_M = \frac{D_m}{D_n} \qquad \alpha = \frac{k_m}{m_\text {thresh} \sigma } \qquad \gamma _M = \frac{\gamma _m}{K_n} \qquad \delta _N = \frac{\delta _n}{\sigma } \qquad \delta _M = \frac{\delta _m}{\sigma }. \end{aligned}$$

In taking $\nu = \sqrt{\frac{\sigma }{D_n}}$ in (5), (6) for simplicity, the dimensionless initial and boundary conditions are:

12 $$\begin{aligned}{} & {} N(X,0) = H(\lambda - X), \end{aligned}$$

13 $$\begin{aligned}{} & {} M(X,0)= {\overline{\eta }}\text { exp}\big (-(X-\lambda )^2\big ), \end{aligned}$$

and

14 $$\begin{aligned} \frac{\partial }{\partial X} N(X,T) = \frac{\partial }{\partial X} M(X,T) = 0 \quad \text {at} \quad X = 0,{\overline{L}}, \end{aligned}$$

where ${\overline{\eta }} = \frac{\eta }{m_\text {thresh}}$ and ${\overline{L}} = L \sqrt{\frac{\sigma }{D_n}}$. We note that the dimensionless values $\lambda , {\overline{\eta }}$ and ${\overline{L}}$ are chosen for illustrative purposes. The corresponding dimensional parameter values are easily computed and are consistent with physiological values.

**Table 1:** Values for the dimensional parameters appearing in (1)–(4)

Parameter	Description	Value	References
$D_n$	Diffusion coefficient of cells	$6.12 \times 10^{-5}$ mm$^2$h$^{-1}$	(Schugart et al. 2008)
$D_m$	Diffusion coefficient of MMPs	$9 \times 10^{-4}$ mm$^2$ h$^{-1}$	(Collier et al. 2011)
$\sigma$	Mitotic rate of cells	0.0385 h$^{-1}$	(Jin et al. 2011)
$K_n$	Carrying capacity of cells	1250 cells mm $^{-2}$	(Jin et al. 2011)
$k_m$	Maximal rate of MMP production by cells	4 pg$/\mu$g h$^{-1}$	Estimate
$\gamma _m$	Density of cells at which half of $k_e$ is attained	625 cell mm $^{-2}$	Estimate
$f_\text {max}$	Maximum factor with which mitosis can be enhanced due to the presence of MMPs	50	Estimate
$\delta _m$	MMP decay rate	0.009 h$^{-1}$	(Deakin and Chaplain 2013)
$\delta _n$	Cell apoptosis rate	0.005 h$^{-1}$	(Jin et al. 2011)
$m_\text {thresh}$	Threshold concentration of MMPs at which maximum healing contribution $f_\text {max}$ is attained	7 pg$/\mu$g	(Muller et al. 2008)

**Table 2:** Values of the dimensionless parameter values appearing in the wound healing model in (9)–(14)

Parameter	Value
$D_M$	14.706
$\alpha$	14.842
$f_\text {max}$	50
$\delta _N$	0.130
$\delta _M$	0.234
$\gamma _M$	0.5
$\lambda$	3
${\overline{\eta }}$	0.1
${\overline{L}}$	200

We define the dimensionless system (9)–(14) as the ‘wound healing model’ and we note that $0\le N(X,T)\le 1$, since we do not expect the cell density to exceed its carrying capacity once healing is completed.

Suitable values for the dimensional parameters appearing in (1)–(4) are given in Table 1; we remark that these parameters correspond to ‘normal’, or healthy, wound healing and, unless otherwise stated, are employed in all simulations. These parameter values are used to calculate the dimensionless parameter values in (11)–(14) and are given in Table 2. We remark that where parameter values were unable to be obtained from literature; these were estimated heuristically.

### Model Simulations

In order to obtain numerical solutions of the wound healing model, we use the method of lines and discretise on a one-dimensional spatial domain, using second order finite differences to approximate spatial derivatives. We then integrate the resulting system of time–dependent ODEs using MATLAB’s ODE15s solver.

**Fig. 3:**
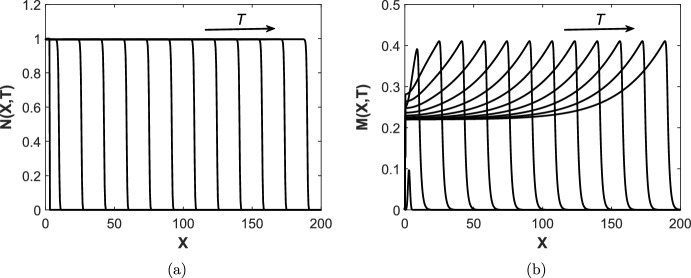
Simulation of the wound healing model (9)–(14) at regular time intervals $T=2$ with parameter values as in Table 2. **a** Evolution of cell density *N*(*X*, *T*) across the spatial domain, **b** Evolution of MMP concentration *M*(*X*, *T*) across the spatial domain

An example numerical simulation of the wound healing model is shown in Fig. 3. As seen in Fig. 3a, the cell density *N*(*X*, *T*) evolves into a travelling wave where a front of tissue of density $N \approx 1$ is ‘invading’ the wound ($N=0$). This state of $N \approx 1$ is close enough to unity to be considered as a healed state; we elaborate on the classification of a healed state further in Sect. 3. Since we observe a travelling wave where a healed front of tissue invades the wound, this scenario corresponds to a wound healing to completion. In Fig. 3b, we also observe the formation of a travelling wave for *M*(*X*, *T*) in which a ‘spike’ in MMP concentration is found at the edge of the healing wound. These qualitative phenomena are in agreement with biological literature, which suggests the increased expression of MMPs at the edge of an acute would at all stages in the healing process (Krejner et al. 2016).

## Steady State and Travelling Wave Analysis

From the previous section, we observe that the wound healing model gives rise to travelling wave solutions. Therefore, in this section we conduct a travelling wave analysis of the model. To determine the far-field states of the travelling waves, we first examine the uniform steady-states of (9), (10). In particular, the invading far-field state for *N*(*X*, *T*) will indicate whether or not a wound is healing to completion. The uniform steady states $({\overline{N}},{\overline{M}})$ of (9), (10) satisfy the following:

15 $$\begin{aligned}{} & {} {\overline{M}} = \frac{\alpha }{\delta _M} \frac{{\overline{N}}(1-{\overline{N}})}{\gamma _M + {\overline{N}}}, \end{aligned}$$

16 $$\begin{aligned}{} & {} \delta _N {\overline{N}} = \big (1 + f_\text {max} {\overline{M}}\text {exp}(1-{\overline{M}})\big )(1-{\overline{N}}){\overline{N}}. \end{aligned}$$

Combining (15), (16), we find that all non-trivial steady states may be written in terms of the single variable ${\overline{N}}$:

17 $$\begin{aligned} \delta _N = \frac{\alpha f_\text {max}(1-{\overline{N}})^2 {\overline{N}}}{\delta _M (\gamma _M+{\overline{N}})} \text {exp}\bigg (1-\frac{\alpha {\overline{N}} (1-{\overline{N}})}{\delta _M (\gamma _M +{\overline{N}})}\bigg ) + 1 - {\overline{N}}. \end{aligned}$$

Using the fimplicit function in MATLAB, we determine a numerical solution for ${\overline{N}}$ and subsequently ${\overline{M}}$ using equation (15).

It is clear from (16) that we always have the trivial steady state, i.e. $({\overline{N}},{\overline{M}})=(0,0)$ for all positive parameters. As discussed in Sect. 2.3, we characterise a wound as healing to completion when a far-field state of $N \approx 1$ is achieved. This is because for $\delta _N=0$, ${\overline{N}}=1$ satisfies (16). For non-zero $\delta _N$, however, ${\overline{N}}$ does not attain the value one, since apoptosis of cells is continually occurring. For small $\delta _N$, i.e. values corresponding to healthy biological functioning, (16) provides ${\overline{N}}$ close to unity, and we hence consider this state to describes healed tissue. Additionally, if an unhealed or partially healed steady-state of ${\overline{N}}$, i.e. not qualitatively close to one, invades a state corresponding to damaged tissue, we characterise this as a chronic wound.

The stability of the uniform steady-states $({\overline{N}},{\overline{M}})$ is given by linear stability analysis of the analogous spatially-independent form of the wound healing model:

18 $$\begin{aligned}{} & {} \frac{dN}{dT} = \big (1 + f_\text {max} M \text {exp}(1-M)\big ) N (1-N) - \delta _N N, \end{aligned}$$

19 $$\begin{aligned}{} & {} \frac{dM}{dT} = \frac{\alpha N}{\gamma _M + N}(1-N) - \delta _M M. \end{aligned}$$

This stability analysis (detailed in Appendix B) is used to determine the stability of the branches of bifurcation diagrams which we examine in Sects. 4 and 6.

In order to verify the existence of travelling solutions to the wound healing model, we consider a travelling wave analysis. Employing the travelling wave coordinates $\xi = X - cT \in {\mathbb {R}}$, where *c* is the wave speed, the wound healing model (9)–(14) reduces to the boundary value problem (BVP):

20 $$\begin{aligned}{} & {} \frac{\textrm{d}^2 N}{\textrm{d} \xi ^2} + c\frac{\textrm{d} N}{\textrm{d} \xi } + \big (1 + f_\text {max} M \text {exp}(1-M)\big ) N (1-N) - \delta _N N = 0, \end{aligned}$$

21 $$\begin{aligned}{} & {} D_M\frac{\textrm{d}^2 M}{\textrm{d} \xi ^2} + c\frac{\textrm{d} M}{\textrm{d} \xi } + \alpha \frac{N}{\gamma _M+N}(1-N) - \delta _M M = 0, \end{aligned}$$

with boundary conditions

22 $$\begin{aligned} \lim _{\xi \rightarrow \pm \infty } N'(\xi ) = \lim _{\xi \rightarrow \pm \infty } M'(\xi ) = 0. \end{aligned}$$

**Fig. 4:**
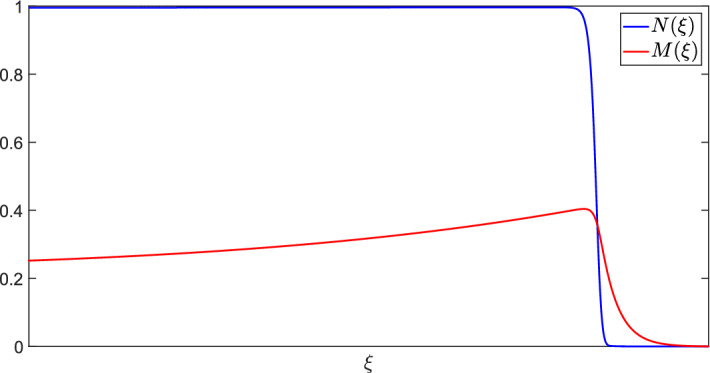
Numerical solution of the boundary value problem (20)–(22) obtained using MATLAB’s BVP5c solver with parameter values as in Table 2

**Fig. 5:**
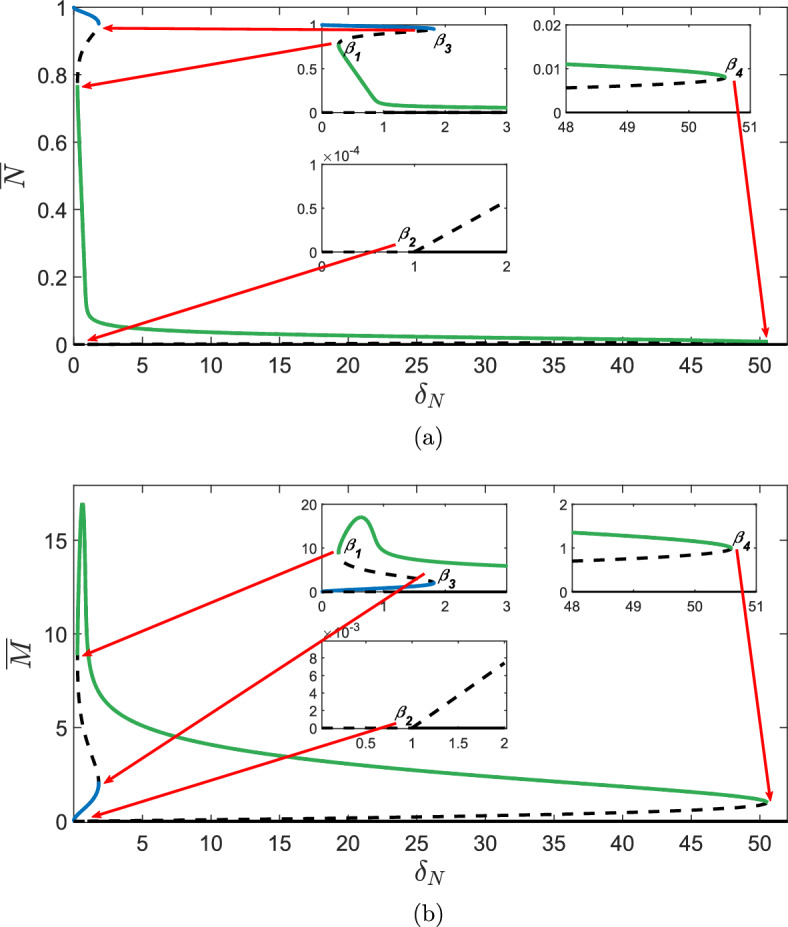
Bifurcation diagrams of (15)–(17) showing the steady-states **a** ${\overline{N}}$ and **b** ${\overline{M}}$ under variation of $\delta _N$. Other parameter values are given in Table 2. The bifurcation diagrams show four bifurcation points $\beta _1, \beta _2, \beta _3$ and $\beta _4$ separating five regimes $I_1, I_2, I_3, I_4$ and $I_5$. Dashed lines represent unstable branches and solid lines represent stable branches. The stable branches are colour-coded such that the green branch of steady states of ${\overline{N}}$ in **a** corresponds to the green branch of steady states of ${\overline{M}}$ in **b**, and similarly for blue branches

We note that when considering solutions in travelling wave coordinates, the stability of ${\overline{N}}$ and ${\overline{M}}$ in the bifurcation diagrams in Figs. 5 and 9 are swapped, i.e. stable branches are unstable in travelling wave coordinates and vice versa, due to the change of variables $\xi =X-cT$.

Using MATLAB’s BVP5c solver, we present numerical simulations of the BVP (20)–(22) in Fig. 4 with parameter values given in Table 2, which verifies the simulations of the wound healing model in Fig. 3. We are also able to use BVP5c to obtain a nondimensional wavespeed of $c=9.6$, which translates to a dimensional wavespeed of $c_\text {dim} = 1.504\times 10^{-2}$mm h$^{-1}$. This wavespeed is of the correct order of magnitude observed from wound healing assays from (Maini et al. 2004a).

## Deregulated Apoptosis

As mentioned previously, a potential cause for the emergence of chronic wounds is deregulated apoptosis. In this section, we examine the behaviour of the wound healing model under variation of the apoptotic rate of cells, $\delta _N$. Figure 5 shows bifurcation diagrams for $\overline{N}$ and $\overline{M}$ against $\delta _N$; the stability of the branches is determined by linear stability analysis that is detailed in Appendix B.

Inspection of the bifurcation diagrams in Fig. 5 show that we have four bifurcation points that separate five parameter regimes, in which solution behaviours of the wound healing model differ qualitatively. The intervals for each regime are denoted as follows:

23 $$\begin{aligned} I_1 = [0, \beta _1); \quad I_2 = (\beta _1,\beta _2); \quad I_3 = (\beta _2,\beta _3);\quad I_4 = (\beta _3,\beta _4) \text { and } I_5 = (\beta _4,\infty ).\nonumber \\ \end{aligned}$$

The positions of the interval boundaries $\beta _1< \beta _2< \beta _3 < \beta _4$ and the method used to calculate them are given in Appendix C. While each interval is discussed in greater detail in subsequent subsections, we begin with a brief overview of the qualitative behaviours observed in each region.

For $\delta _N \in I_1$, we have two steady-states for ${\overline{N}}$: a state approaching unity and the trivial state. As discussed in Sect. 2.3, this parameter regime corresponds to a wound healing to completion, with an increased expression of MMPs at the wound edge. For $\delta _N \in I_2, I_3$, it is unclear from the bifurcation diagram whether or not a wound will heal to completion, due to the existence of other non-trivial steady states. The bifurcation diagram for ${\overline{N}}$ indicates that we have 3 and 4 nontrivial states for $I_2$ and $I_3$ respectively: with one being a healed state (${\overline{N}} \approx 1$), and the others being partially healed states. At $\delta _N=\beta _3$, we observe a saddle node bifurcation point at which the healed state is destroyed. For $\delta _N \in I_4$, there are two non-trivial states for ${\overline{N}}$, both being unhealed states which indicates that for $\delta _N \in I_4$, a wound will not heal to completion and hence a chronic wound will persist. Finally, for $\delta _N \in I_5$ only the trivial state exists.

### $\delta _N \in I_1$

**Fig. 6:**
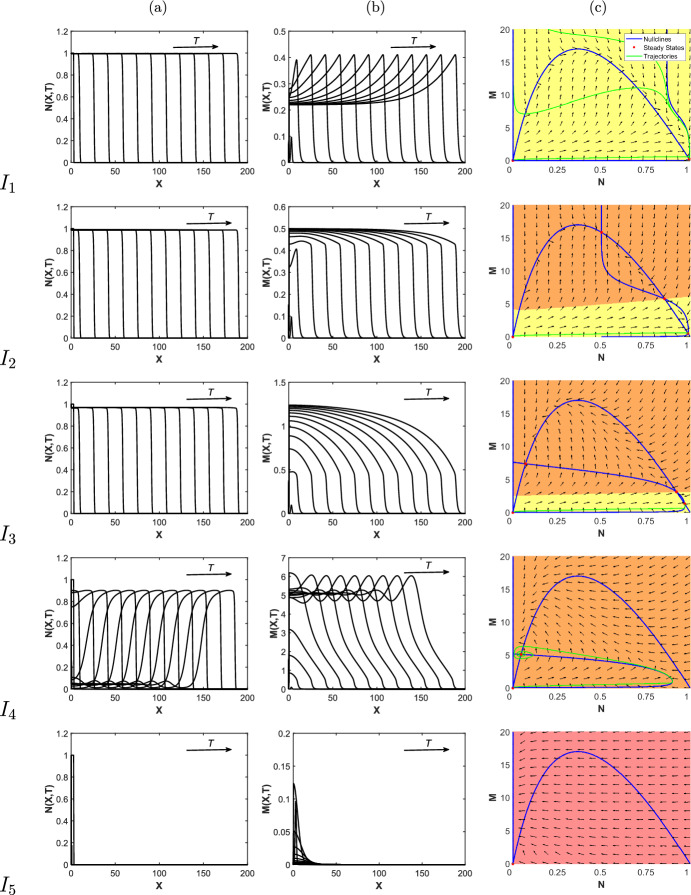
Direct simulations of the wound healing model, for various $\delta _N$ values for **a** *N*(*X*, *T*) and **b** *M*(*X*, *T*). The phase plane diagrams in **c** correspond to the spatially independent ODE system (18), (19). Blue lines represent nullclines, green lines indicate representative trajectories of the ODE system and red dots represent the steady states. The yellow highlighted section of the domain represents the set of all the initial conditions (ICs) in the (*N*, *M*) phase space whose trajectories go to the healed state, the orange section represents the ICs whose trajectories go to an unhealed state and the red section represents the ICs whose trajectories go to the zero state. All other parameter values are as given in Table 2. We note that for $I_2$ and $I_3$, only the fully healed travelling waves (obtained using initial conditions as in (12), (13)) are shown in **a** and **b**. We also note that the *M*-axis changes in each row

The bifurcation diagram in Fig. 5 indicates that we have two steady-state solutions, with one being the trivial state. The other state is ${\overline{N}} \sim 1$ at leading order, which can be verified using a regular perturbation analysis by considering the case where $\delta _N$, $\delta _M$ $\ll 1$. As discussed in Sect. 3, this regime describes the invasion of a wound by a healed front of cells and thus the healing of an acute wound. These features are reflected in direct simulation of the full system in Fig. 6a, for which $\delta _N \in I_1$. We also note that travelling waves for *M*(*X*, *T*) in Fig. 6b display an increased expression of MMP concentration at the wound edge, which is in agreement with the biological literature as discussed in Sect. 1. From the phase plane diagram in Fig. 6c, we see that regardless of initial conditions, trajectories in (*N*, *M*) space always arrive at the healed state, $({\overline{N}},{\overline{M}})$. Therefore, for $\delta _N \in I_1$ and other parameter values as in Table 2, a wound will always heal to completion.

### $\delta _N \in I_2,I_3$

For $\delta _N \in I_2, I_3$, the bifurcation diagram in Fig. 5 shows that we have 3 and 4 nontrivial states, respectively. The phase plane diagrams in Fig. 6c indicate that the initial state determines whether or not the wound heals to completion, with high initial concentrations of MMPs resulting in the emergence of a chronic wound. Contrastingly, if a small concentration of MMPs is initially present at the wound edge, a wound can heal to completion (Fig. 6a). This suggests that baseline levels of MMP must be kept low in order to minimise risk of the persistence of a chronic wound. The simulations for $\delta _N \in I_2, I_3$ exhibit behaviour similar to that for which $\delta _N \in I_1$, demonstrating the healing of an acute wound.

### $\delta _N \in I_4$

For $\delta _N \in I_4$, all trajectories arrive at one of two unhealed states for all initial conditions (Fig. 6c). These states have complex eigenvalues, characterised by spirals in the phase plane diagram and we therefore expect a travelling wave corresponding to a chronic wound with fluctuating MMP levels about its nonzero state. This is verified by the simulations in Fig. 6a, b, where we observe travelling waves with qualitatively different features to those observed in previously discussed intervals.

This change in the qualitative features of the travelling waves corresponds to a saddle-node bifurcation point at $\delta _N=\beta _3$ observed in the bifurcation diagrams in Fig. 5. At this bifurcation point, the branch of healed states ${\overline{N}} \sim 1$, shown in blue (and the corresponding branch for ${\overline{M}}$) is destroyed. This loss of a healed state of ${\overline{N}}$ indicates that the invading state in the travelling wave simulations will transition to an unhealed state, which is verified by Fig. 6a where we also observe increased density of dermal tissue at the edge of the wound. As discussed in Sect. 3, we characterise this as a chronic wound. The emergence of a chronic wound also corresponds to the invading state of ${\overline{M}}$ in the travelling wave simulations transitioning to a larger value as shown in Fig. 6b, suggesting that elevated MMP concentrations prevent the complete healing of the wound. These results are consistent with the biological literature in that deregulated apoptosis is a prominent cause of the emergence of chronic wounds, which are characterised by having raised MMP concentrations.

### $\delta _N \in I_5$

For $\delta _N \in I_5$, we find that *N* and *M* both tend to zero for all initial conditions. This collapse of the travelling wave is expected in the case where apoptosis of dermal cells is very high, as cells die faster than they can reproduce.

## Multistability and Hysteresis

**Fig. 7:**
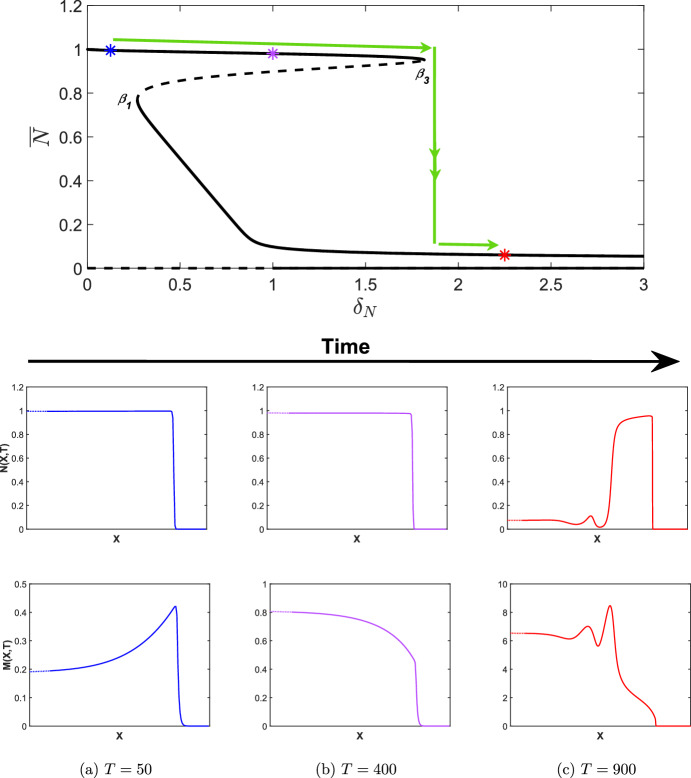
Simulations of the wound healing model with (24) applied to (9), i.e. $\delta _N$ increasing in time with $\widehat{\delta _N}=0.1$ and $\epsilon =0.01$. Other parameter values are as given in Table 2. Note that the *X*-axis has been trimmed to highlight the qualitative features of each travelling wave (dotted lines) but has not been scaled, i.e. the *X*-axis is the same length scale as those in Fig. 6a and b. The bifurcation diagram is as given in Fig. 5a for $\delta _N \in [0,3]$ and coloured asterisks represent the $\delta _N(T)$ values at the time points given in **a**, **b** and **c**. The green arrows represent the evolution of the invading states of the travelling waves as $\delta _N$ is increased. Note that all travelling waves shown connect to the zero state. We also note that the *M*-axis changes at each time point

**Fig. 8:**
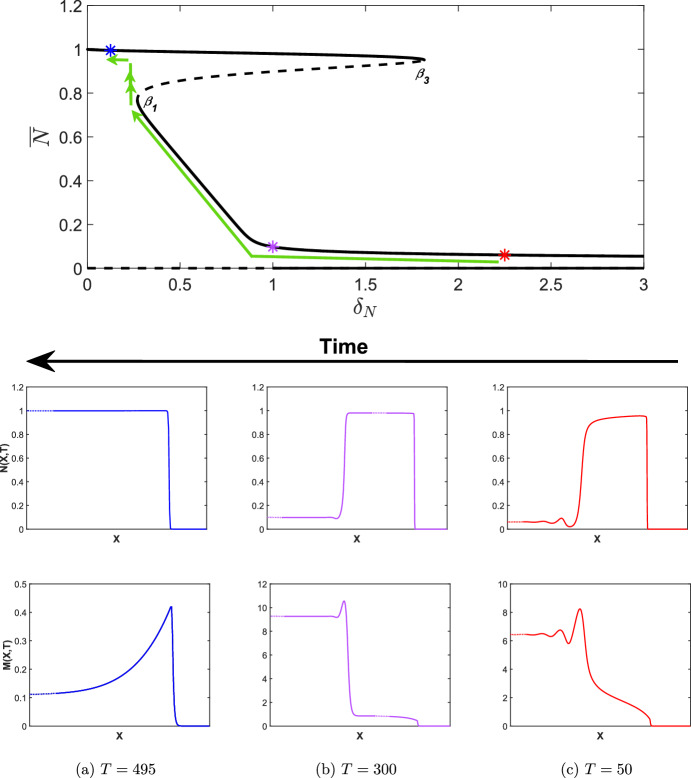
Simulations of the wound healing model with (24) and $\epsilon \rightarrow -\epsilon$ applied to (9), i.e. $\delta _N$ decreasing in time with $\widehat{\delta _N}=2.5$ and $\epsilon =0.005$. Other parameter values are as given in Table 2. Note that the *X*-axis has been trimmed to highlight the qualitative features of each travelling wave (dotted lines) but has not been scaled, i.e. the *X*-axis is the same length scale as those in Fig. 6a, b. The bifurcation diagram is as given in Fig. 5a for $\delta _N \in [0,3]$ and coloured asterisks represent the $\delta _N(T)$ values at the time points given in **a**, **b** and **c**. The green arrows represent the evolution of the invading states of the travelling waves as $\delta _N$ is increased. Note that all travelling waves shown connect to the zero state. We also note that the *M*-axis changes at each time point

As discussed in Sect. 1, a potential cause for the emergence of chronic wounds is deregulated apoptosis which may be caused by uncontrolled blood sugar levels in diabetes patients. In this section, we therefore examine how the apoptotic rate, $\delta _N$ controls the transition from a healthy to a chronic state. We also investigate whether or not a chronic wound may be reversed if the apoptotic rate were to be regulated, for example, by regulating blood sugar levels. As discussed in Sect. 4.3, a saddle-node bifurcation point occurs at $\delta _N = \beta _3$, which results in the emergence of a chronic wound, where high concentrations of MMPs prevent the wound healing to completion. The subcritical nature of this bifurcation has important implications for the solution behaviour, which we demonstrate in direct simulations of the wound healing model by allowing $\delta _N$ in (9) to slowly increase in time:

24 $$\begin{aligned} \delta _N(T) = \widehat{\delta _N} + \epsilon T, \end{aligned}$$

where $\widehat{\delta _N} = 0.1 \in I_1$. As we see in Fig. 7, the travelling wave for *N*(*X*, *T*) transitions from a completely healed wound to a chronic wound with elevated MMP concentrations, which verifies the results outlined in Sect. 4.3.

As mentioned in Sect. 4.3, a saddle-node bifurcation point also occurs at $\delta _N=\beta _1$. As such, we consider the case of decreasing $\delta _N$ to investigate the implications for the solution behaviour upon decreasing $\delta _N$ below $\beta _1$ from a regime in which a chronic wound persists. We take a value of $\widehat{\delta _N}$ such that $\widehat{\delta _N} > \beta _3$ (to ensure the persistence of a chronic wound), and allow $\delta _N$ in (9) to slowly decrease in time via (24) by changing $\epsilon$ with $-\epsilon$. In Fig. 8, we take $\widehat{\delta _N} = 2.5 \in I_4$ and simulate the wound healing model with $\delta _N(T)$ applied to (9). From Fig. 8c, we observe the emergence of a chronic wound as we expect, displaying consistent qualitative features as in Fig. 7c when considering increasing $\delta _N$. For $\beta _1<\delta _N<\beta _3$, i.e. $\delta _N \in I_2, I_3$, however, we observe another partially-healed wound in the travelling wave simulations in Fig. 8b in contrast to Fig. 7b and demonstrates the existence of two qualitatively different travelling wave profiles, depending on the choice of initial conditions. We observe from Fig. 8a that for a chronic wound to heal, $\delta _N(t)$ must be decreased to when $\delta _N < \beta _1$, i.e $\delta _N \in I_1$, demonstrating the subcritical nature of the bifurcation point $\beta _1$, and the multistable nature of different travelling wave profiles for $\beta _1< \delta _N < \beta _3$. The multistability of the travelling wave profiles occurs as there is history dependence upon increasing and decreasing the apoptotic rate, i.e. a hysteresis loop affects the invading states of the travelling waves profiles. We conclude that chronic wounds may persist if apoptotic rates reach an unregulated state, but these chronic wounds may heal to completion if $\delta _N$ were able to be regulated from a deregulated state, for example by controlling blood sugar levels.

## Elevated MMP Production

**Fig. 9:**
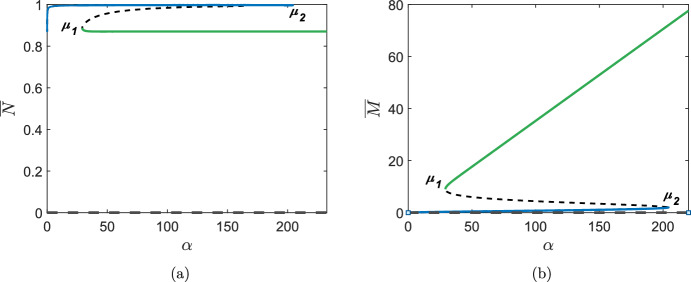
Bifurcation diagrams of $\alpha$ against **a** ${\overline{N}}$, and **b** ${\overline{M}}$. Other parameter values are as given in Table 2. Dashed lines represent unstable branches and solid lines represent stable branches. The stable branches are colour-coded such that the green branch of steady states of ${\overline{N}}$ in **a** correspond to the green branch of steady states of ${\overline{M}}$ in **b**; equivalent for blue branches

As described in Sect. 1, chronic wounds are characterised by elevated levels of MMPs which prevent its healing. MMP production is a factor that may potentially be targeted with treatments for chronic wounds. In a similar fashion to the varying of $\delta _N$, we now examine the qualitative changes to travelling wave profiles when we vary the production rate of MMPs, $\alpha$.

We present bifurcation diagrams for ${\overline{N}}$ and ${\overline{M}}$ against $\alpha$ in Fig. 9; the stability of the branches is determined by linear stability analysis detailed in Appendix B. Inspection of the bifurcation diagrams show that we have two saddle-node bifurcation points ($\mu _1$ and $\mu _2$) separating three parameter regimes. We note that at $\mu _2$ (at which $\alpha$ is large), the branch of healed states of ${\overline{N}}$ is destroyed and hence, similar to in Sect. 4.3, we expect the emergence of a chronic wound. We also note that the unhealed branch in Fig. 9 consists of states much higher than those in Fig. 5 upon varying $\delta _N$. This contrast is due to our choice of *f*(*m*) in (3). If we allowed *f*(*m*) to take negative values for large *m*, then the unhealed branch would take much lower values.

**Fig. 10:**
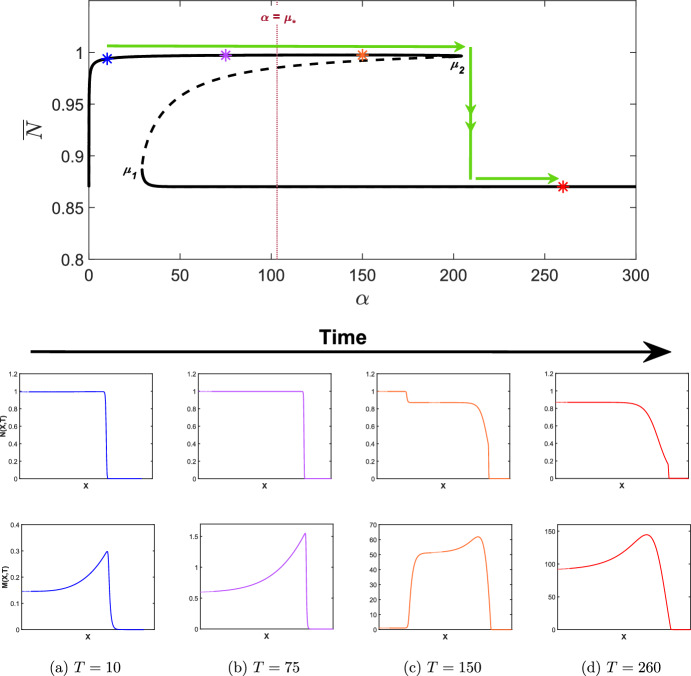
Simulations of the wound healing model with (25) applied to (10), i.e. $\alpha$ increasing in time with ${\widehat{\alpha }}=0$ and $\epsilon =1$. Other parameter values are as given in Table 2. Note that the *X*-axis has been trimmed to highlight the qualitative features of each travelling wave (dotted lines) but has not been scaled, i.e. the *X*-axis is the same length scale as those in Fig. 6a and b. The bifurcation diagram is as given in Fig. 9a for $\alpha \in [0,300]$ and ${\overline{N}} \in [0.8,1]$ and coloured asterisks represent the $\alpha (T)$ values at the time points given in **a**, **b**, **c** and **d**. The green arrows represent the evolution of the invading states of the travelling waves as $\alpha$ is increased. The maroon dotted line represents the value $\alpha =\mu _{*}$. Note that all travelling waves shown connect to the zero state. We also note that the *M*-axis changes at each time point

Similar to Sect. 5, we now investigate the dynamics of the wound healing model for both increasing and decreasing $\alpha$. Firstly, we allow $\alpha$ in (10) to slowly increase in time:

25 $$\begin{aligned} \alpha (T) = {\widehat{\alpha }} + \epsilon T, \end{aligned}$$

where ${\widehat{\alpha }} = 0 < \mu _1$. As we see in Fig. 10, the travelling wave for *N*(*X*, *T*) transitions from a completely healed wound to a partially healed wound with elevated MMP concentrations (see the bifurcation diagram in Fig. 9b). These dynamics occur as $\alpha$ exceeds a value $\mu _* \approx 103.2$ determined heuristically, where, despite the invading state of the travelling wave attaining a healed value, the wave visits the unhealed state, resulting in a partially healed wound. Increasing $\alpha$ further results in the invading state transitioning to an unhealed value, and thus the emergence of a chronic wound at $\mu _2$. We deduce that the threshold MMP production rate at which wounds cease to heal to completion is $\mu _{*}$, rather than the saddle-node bifurcation point $\mu _2$ as we may have originally expected.

**Fig. 11:**
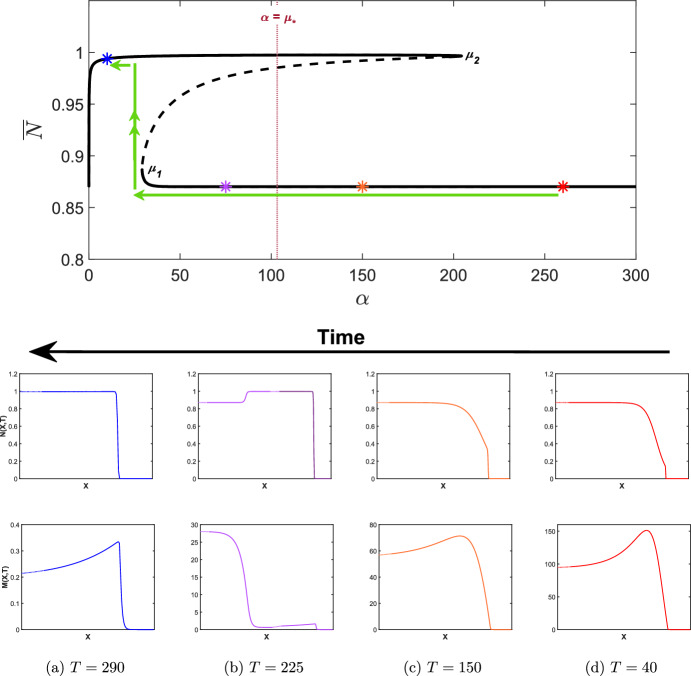
Simulations of the wound healing model with (25) and $\epsilon \rightarrow -\epsilon$ applied to (10), i.e. $\alpha$ decreasing in time with ${\widehat{\alpha }}=300$ and $\epsilon =1$. Other parameter values are as given in Table 2. Note that the *X*-axis has been trimmed to highlight the qualitative features of each travelling wave (dotted lines) but has not been scaled, i.e. the *X*-axis is the same length scale as those in Fig. 6a and b. The bifurcation diagram is as given in Fig. 9a for $\alpha \in [0,300]$ and ${\overline{N}} \in [0.8,1]$ and coloured asterisks represent the $\alpha (T)$ values at the time points given in **a**, **b**, **c** and **d**. The green arrows represent the evolution of the invading states of the travelling waves as $\alpha$ is decreased. The maroon dotted line represents the value $\alpha =\mu _{*}$. Note that all travelling waves shown connect to the zero state. We also note that the *M*-axis changes at each time point

We now consider decreasing $\alpha$ to investigate whether or not a chronic wound may be reversed if MMP production levels were able to be regulated. In Fig. 11, we take $\alpha =300>\mu _2$ and allow $\alpha$ in (10) to slowly decrease in time via (25) by changing $\epsilon$ with $-\epsilon$. From Fig. 11a, we observe the emergence of a chronic wound as we expect, showing consistent qualitative features as in Fig. 10d when considering increasing $\alpha$. For $\mu _1<\alpha <\mu _2$ however, we observe wounds that do not heal to completion in contrast to the case of increasing $\alpha$, again demonstrating the existence of multistable travelling wave profiles depending on the choice of initial conditions. We also observe a change in qualitative features at $\alpha =\mu _*$. For $\alpha < \mu _*$, the travelling wave visits the healed state resulting in a partially healed wound. From Fig. 11d, we deduce that $\alpha (T)$ must be reduced such that $\alpha < \mu _1$ for a chronic wound to heal.

Similar to in Sect. 5, there is a hysteresis loop which results in history dependence of the travelling wave profiles on increasing and decreasing $\alpha$ which results in multistable travelling wave profiles for $\mu _1< \alpha < \mu _2$. Unlike in Sect. 5 however, we observe an inconsistency in qualitative features of travelling waves for $\mu _1< \alpha < \mu _2$ due to the existence of the threshold value $\mu _*$. We conclude that that wounds will only partially heal beyond $\alpha =\mu _*$. Furthermore, a chronic wound may be reversed to a healed state if MMP production rate is controlled from a deregulated state back to a regulated state.

## Conclusions and Discussion

In this work, we develop a two-variable reaction-diffusion model, describing the interaction of matrix metalloproteinases (MMPs) with dermal cells in the wound healing process. In particular, we focus attention on the emergence of chronic wounds, since biological literature suggests that elevated levels of MMPs play a key role in their emergence. Our mathematical model gives rise to travelling wave solutions and in particular is able to emulate key qualitative features of the wound healing process reported in biological literature. One such property is that under parameter regimes representing healthy biological functioning, we observe acute wounds healing to completion with an increased expression in MMP concentration at the edge of the healing wound.

To contrast acute wounds with chronic wounds, we consider the effect of varying the apoptotic rate of dermal cells. Small apoptotic rates correspond to healthy biological functioning and thus we observe the complete healing of a wound. Provided initial concentrations of MMPs are kept low, an increase in apoptotic rate also results in the healing of a wound, which suggests that in order to avoid risk of the emergence of a chronic wound, baseline levels of MMPs must be kept minimal. Elevated apoptotic rate beyond a threshold value leads to the emergence of a chronic wound, with increased levels of MMPs invading the wound which prevents its healing. Moreover, we observe mulitstable travelling waves depending on initial conditions due to the existence of a hysteresis loop in the bifurcation diagrams for apoptotic rate. Therefore, in order to reverse a chronic wound to a state of complete healing, the apoptotic rate must be decreased below a threshold value. This gives insights into the regulation of apoptotic rate in the healing of chronic wounds which may be achieved by, for example, regulating blood sugar levels in diabetes patients.

We also consider the effect of varying MMP production rate on the healing of a wound. Similar to the analysis for apoptotic rate, we obtain threshold values at which chronic wounds persist and may be reversed. However, contrasting from the variation of the apoptotic rate, we observe a change in the the qualitative features of the travelling wave solutions at a different threshold value $\mu _{*}$. At this new threshold, occurring in the middle of the multistable regime, a wound transitions from healing completely to only being partially healed, before reaching a regime where chronic wounds persist.

Due to the observation of multistable travelling wave solutions with varying qualitative features, a natural extension to this work includes conducting a stability analysis of these travelling waves. Moreover, since we have considered the effects of varying apoptotic rate and MMP production rate separately, future work should consider the impact of concurrent defects in apoptotic rate and MMP production levels on the healing of a wound. We note that we have defined a chronic wound by changes to apoptotic rates and MMP production rates starting from a parameter set corresponding to acute wound healing. Parameter values corresponding to a chronic wound may however involve additional changes beyond these, hence further work should consider a full parameter sensitivity analysis.

Despite the wound healing model successfully being able to emulate qualitative features of both acute and chronic wounds, it has greatly simplified the process of wound healing by coupling the action of fibroblasts and the extracellular matrix, as well as assuming that MMPs directly contribute to healing of wounds. In reality, MMPs indirectly contribute to the healing by allowing the migration of other key elements involved in the wound healing process, such as fibroblasts, keratinocytes and endothelial cells (Nyugen et al. 2016). Future research should therefore take these factors into consideration. Furthermore, the role of tissue inhibitors of MMPs (TIMPs) in the wound healing process should be considered as these have an impact on the regulation of MMP concentration. This consideration would ultimately give further insight into the behaviour of MMPs during the wound healing process, as well as potentially direct biological therapies of chronic wounds, i.e. by determining the optimum physical and chemical composition of hydrogel therapies.

## Figures and Tables

**Fig. 12:**
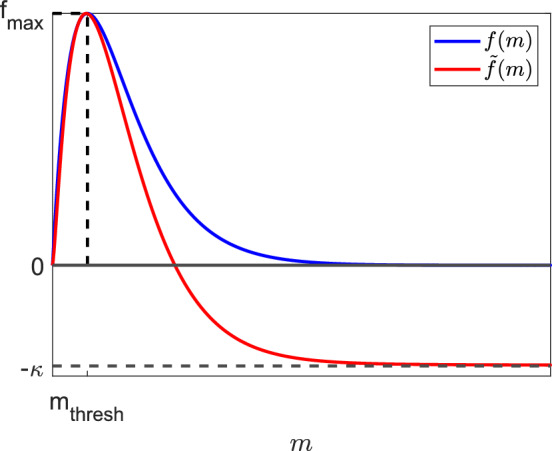
Schematic functional forms of the healing contribution by MMPs functions *f*(*m*) as in Fig. 2a and ${\tilde{f}}(m)$ as in (26)

**Fig. 13:**
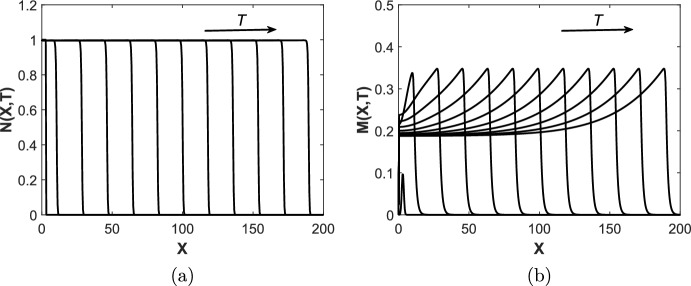
Simulation of the system (1)–(7), non-dimensionalised according to (8) with *f*(*m*) as in (26) at regular time intervals with ${\tilde{f}}_\text {max}=67.46$, $\kappa =20$ and other parameter values as in Table 2. **a** Evolution of cell density *N*(*X*, *T*) across the spatial domain, **b** Evolution of MMP concentration *M*(*X*, *T*) across the spatial domain

## Appendix A: Alternative *f*(*m*)

In Sect. 2, we consider the functional form of *f*(*m*) in (1) which describes the healing contribution of dermal cells by MMPs. Since large concentrations of MMPs results in the destruction of the ECM, we may expect *f*(*m*) to become negative for large *m*. One such function that captures this behaviour is the following:

26 $$\begin{aligned} {\tilde{f}}(m) = \frac{{\tilde{f}}_\text {max}}{m_\text {thresh}} m \text { exp}\bigg (1-\frac{m}{m_\text {thresh}}\bigg ) - \frac{\kappa m}{1+m}, \end{aligned}$$

for some values of ${\tilde{f}}_\text {max}$ and $\kappa$. A schematic of ${\tilde{f}}(m)$ is shown in Fig. 12. In non-dimensionalising the PDE system (1), (2) subject to ${\tilde{f}}(m)$ given in (26), using the scaling given in (8), we obtain the simulations given in Fig. 13. Initial and boundary conditions are chosen as in (12)–(14) and we take ${\tilde{f}}_\text {max}=67.46$ and $\kappa =20$; all other parameter values are as given in Table 2. In Fig. 13, we observe largely similar qualitative features as those seen in Fig. 3: the invasion of a wound by a healed front of cells with an increased expression of MMP concentration at the wound edge.

## Appendix B: Steady State Stability Analysis

In this section, we consider the stability of spatially-uniform steady states that solve (18), (19). We note that the Jacobian of this system is given by:

27 $$\begin{aligned} J = \begin{pmatrix} (1-2N) \big (1 + f_\text {max} M \text {exp}(1-M)\big ) - \delta _N &{} \quad f_\text {max}N(1-N)(1-M) \big (\text {exp}(1-M)\big )\\[6pt] \dfrac{\alpha (\gamma _M-N^2-2\gamma _M N)}{(\gamma _M+N)^2} &{} -\delta _M \end{pmatrix}.\nonumber \\ \end{aligned}$$

At the steady state $({\overline{N}},{\overline{M}})=(0,0)$, the Jacobian becomes

$$J|_{(0,0)}=$$ $$ \begin{pmatrix} 1-\delta _N &{} 0\\[6pt] \dfrac{\alpha }{\gamma _M} &{} -\delta _M \end{pmatrix},$$

which provides the following eigenvalues:

28 $$\begin{aligned} \begin{aligned} \lambda _1&= -\delta _M, \\ \lambda _2&= 1-\delta _N. \end{aligned} \end{aligned}$$

Since $\delta _M, \delta _N >0$, the zero state is an unstable saddle for $\delta _N<1$ and is a stable node for $\delta _N>1$. For all other steady states, we use MATLAB’s fsolve function to determine $({\overline{N}},{\overline{M}})$ which are then substituted into (27) and whose eigenvalues are computed using MATLAB’s eig function.

## Appendix C: Bifurcation Points $\delta _N$

From the bifurcation diagrams in Fig. 5, we observe that there are four bifurcation points: one transcritical and three saddle nodes. The value of $\delta _N$ at which the transcritical bifurcation point occurs may be deduced from the argument in Appendix B. From (28), we conclude that the zero state is an unstable saddle for $\delta _N<1$ and is a stable node for $\delta _N>1$. This change in stability confirms that the transcritical bifurcation point occurs at $(\delta _N,{\overline{N}},{\overline{M}})=(1,0,0)$.

The values of ${\overline{N}}$ at which the saddle node bifurcation points occur may be calculated by computing the derivative of (17) with respect to ${\overline{N}}$ and setting this equal to zero, i.e.

29 $$\begin{aligned}{} & {} \frac{\alpha f_\text {max}}{\delta _M (\gamma _M + {\overline{N}})^2}\bigg (\gamma _M(1-4{\overline{N}}+3{\overline{N}}^2)-2{\overline{N}}^2(1-{\overline{N}}) \nonumber \\{} & {} \qquad + \frac{\alpha {\overline{N}} (1-{\overline{N}})^2(2\gamma _M {\overline{N}} - \gamma _M + {\overline{N}}^2)}{\delta _M (\gamma _M + {\overline{N}})}\bigg ) \text {exp}\bigg (1-\frac{\alpha {\overline{N}} (1-{\overline{N}})}{\delta _M (\gamma _M +{\overline{N}})}\bigg ) + 1 = 0.\nonumber \\ \end{aligned}$$

We use MATLAB’s fzero function to find the ${\overline{N}}$ values of the saddle node bifurcation points, and then substitute these into (15) and (17) to find the corresponding ${\overline{M}}$ and $\delta _N$ values respectively. For the parameter choices as in Table 2, the saddle-node bifurcation points are $(\delta _N,{\overline{N}},{\overline{M}}) = (0.2695,0.7693,8.8787)$, (1.8164, 0.9508, 2.0465) and (50.5904, 0.007998, 0.9916). I.e. the values of $\beta _1, \beta _2, \beta _3$ and $\beta _4$ as discussed in Sect. 4, subject to other parameter values as in Table 2, are $\beta _1 = 0.2965, \beta _2 = 1, \beta _3 = 1.9164$ and $\beta _4 = 50.5904$.
